# A novel Pyroptosis‐related long non‐coding RNA signature for predicting the prognosis and immune landscape of head and neck squamous cell carcinoma

**DOI:** 10.1002/cam4.4819

**Published:** 2022-05-14

**Authors:** Chongchang Zhou, Yiming Shen, Yangli Jin, Zhisen Shen, Dong Ye, Yi Shen, Hongxia Deng

**Affiliations:** ^1^ Department of Otorhinolaryngology Head and Neck Surgery Ningbo Medical Center Lihuili Hospital Ningbo China; ^2^ Department of Otorhinolaryngology Head and Neck Surgery Lihuili Hospital affiliated to Ningbo University Ningbo China; ^3^ Department of Ultrasonography Ningbo Yinzhou Second Hospital Ningbo China

**Keywords:** head, immunotherapy, long noncoding RNA, neck squamous cell carcinoma, prognosis, pyroptosis

## Abstract

**Background:**

Pyroptosis plays an essential function in carcinogenesis and the antitumor immune response. Herein, we constructed a pyroptosis‐related long noncoding RNA (prLncRNA) signature to predict therapeutic effects and outcomes for head and neck squamous cell carcinoma (HNSCC) patients.

**Methods:**

Patients obtained from the TCGA‐HNSC project were divided randomly into the training as well as the validation sets at a ratio of 7:3. A novel prognostic prLncRNA signature was constructed from the results of the training set using the least absolute shrinkage and selection operation. The medium value was used as the basis for categorizing all HNSCC patients into a low‐ or high‐risk cohort. Cox regression and Kaplan–Meier (KM) survival analyses were executed to estimate the prognostic value. We also evaluated the functional enrichment, tumor microenvironment, immune cell infiltration, and the sensitivity to chemotherapy and immunotherapy between the high‐ and low‐risk cohorts.

**Results:**

Nineteen prognostic prlncRNAs were identified to establish the prognostic signature. Multivariate Cox regression and KM survival analyses confirmed that this prlncRNA signature might serve as an independent prognostic indicator of patient survival, which was subsequently confirmed using a validating dataset. Multiple ROC curves indicated the prlncRNA signature presented a more predictive power than clinicopathological factors (age, sex, tumor grade, and tumor stage). GO, KEGG, and GSEA enrichment analysis disclosed several immune‐related pathways which appeared to be enhanced in the low‐risk cohort. ESTIMATE, CIBERSORT, and ssGSEA algorithms indicated considerable differences in the tumor microenvironment and immune cell infiltration in the low‐ and high‐risk cohorts. Furthermore, the low‐risk cohort was predicted to achieve a better response to immunotherapeutic drugs, while in contrast, the high‐risk cohort would be more sensitive to chemotherapy drugs.

**Conclusions:**

Our findings robustly demonstrate that our constructed prlncRNA signature could serve as an efficient indicator of prognosis, immunotherapy response, and chemosensitivity for HNSCC patients.

## Graphical abstract

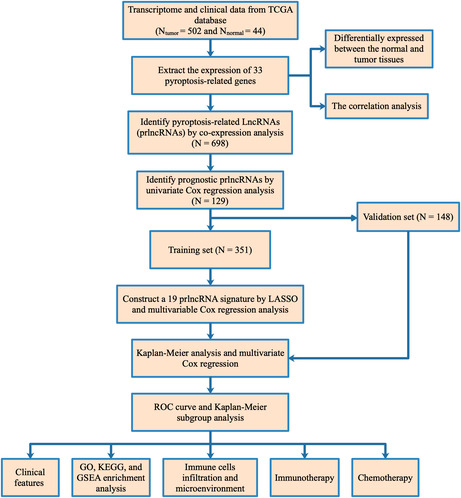

## 1 INTRODUCTION

According to epidemiological statistics, head and neck cancers rank sixth among malignant tumors globally with nearly 650,000 new incidents and 380,000 fatalities reported worldwide annually.^1^ Approximately 90% of the pathological types of head and neck cancers are squamous cell carcinomas (HNSCC). Cigarette use and alcohol consumption are generally considered to be risk factors. However, the incidence of human papillomavirus (HPV)‐related HNSCC has increased sharply over the past two decades,^2^, ^3^ which has altered the epidemiologic distribution, the distribution of affected anatomical subsites, and survival outcomes.^4^ Due to the lack of effective screening tools and early clinical symptoms, the majority of HNSCC patients are often at an advanced disease stage by the time they are diagnosed, which results in a dismal 5‐year survival rate that is below 50%.^5^ Thus, finding an effective and reliable method for risk stratification, guiding treatment, and improving the prognosis of HNSCC patients is urgently required.

Pyroptosis, which has recently been identified as inflammation‐related programmed cell death, involving two main molecular signaling pathways, namely caspase‐1‐dependent canonical as well as caspase‐4/5/11‐dependent noncanonical inflammasome pathways.^6^ Morphologically different from apoptosis, pyroptosis has been ordinarily referred to as cell swelling and lysis, which results in the production of pro‐inflammatory mediators interleukin (IL)‐1β, IL‐18, and cellular contents into the extracellular space and activation of the inflammatory response.^7^, ^8^ Recently, extensive investigations have shown that pyroptosis has become a hot topic implicated in the treatment approach in numerous illnesses^9^, ^10^, ^11^ and especially, in malignant tumors. In general, pyroptosis may play both tumor‐supporting and tumor‐suppressing roles during the development of cancers. On one hand, pyroptosis leads to the release of inflammatory mediators, which might promote cancer initiation and progression.^12^, ^13^ On the other hand, pyroptosis, as a form of cell death, and has been linked to tumor suppression, which makes it an attractive new therapeutic indication for cancer treatment.^14^ Increasing evidence has suggested that pyroptotic cell death represents a novel killing mechanism involved in the antitumor therapeutic effects of chemotherapy and immunotherapy.^15^, ^16^, ^17^

Recently, some studies have found that long noncoding RNAs (lncRNAs) are involved in more complex biological processes than initially anticipated and have been functionally correlated with multiple diseases, and in particular cancers.^18^ The regulatory function of lncRNAs participates in numerous cellular activities such as cell growth, cell proliferation, viability, apoptosis, angiogenesis, evasion of tumor suppressors, and metastatic processes.^19^, ^20^ To date, there have been very few studies on pyroptosis‐related lncRNAs. LncRNAs are broadly involved in the pathological processes of diverse diseases and regulate pyroptosis signaling pathway‐related proteins through miRNAs.^21^ LncRNA MEG3 induces renal tubular epithelial cell pyroptosis through the regulation of the miR‐18a‐3p/GSDMD pathway in lipopolysaccharide‐induced acute kidney injury.^22^ LncRNA RP1‐85F18.6 remarkably promotes pyroptosis in colorectal cancer cells by cleaving GSDMD.^23^ Nevertheless, to the best of our knowledge, pyroptosis‐related lncRNAs (prLncRNAs) have not been studied in patients with HNSCC.

In the current research, we obtained pyroptosis‐related lncRNAs from the Cancer Genome Atlas (TCGA) database by Pearson correlation analysis. Next, we constructed a pyroptosis‐related lncRNA signature with 19 prognostic lncRNAs for HNSCC using both the Least Absolute Shrinkage and Selection Operator (LASSO) regression and Cox proportional hazard regression analysis. We also evaluated the prognostic value of signature and the association with the tumor microenvironment, infiltrating immune cells, and response to immunotherapy and chemotherapeutics.

## 2 MATERIALS AND METHODS

### 2.1 Patient data

The transcriptome profiles, represented as the fragment per kilobase million (FPKM) values from the TCGA‐HNSC project, included 502 HNSCC and 44 normal tissue specimens were retrieved from TCGA database (https://portal.gdc.cancer.gov/, updated January 6, 2021). The corresponding clinicopathologic information (including clinical stage, age, sex, grade, T‐stage, N‐stage, overall survival [OS] time, and status) were also extracted using the same method. Only 499 cases with corresponding prognostic information were used for the next analysis.

### 2.2 Expression of pyroptosis‐related genes and association with HNSCC

A total of 33 genes previously reported to be associated with pyroptosis were identified by literature mining and are reported in Table S1.^10^, ^24^, ^25^, ^26^, ^27^, ^28^, ^29^, ^30^, ^31^, ^32^, ^33^, ^34^, ^35^, ^36^ The expression of these pyroptosis‐related genes was extracted and was contrasted in HNSCC and normal tissue using the “limma” package. An interaction network for pyroptosis‐related genes was constructed with |Pearson *R*| > 0.2 and *p* < 0.05 using the “igraph” and “reshape2” packages.

### 2.3 Development of the pyroptosis‐related lncRNAs prognostic signature and risk model

We downloaded the human gene transfer format (GTF) file from the Ensembl dataset (http://asia.ensembl.org) for annotation of gene IDs to identify mRNAs and lncRNAs. Pearson correlation analysis was executed for pyroptosis‐related genes and all lncRNAs to identify pyroptosis‐related lncRNAs (|Pearson *R*| > 0.4 and *p* < 0.001). Next, univariate Cox regression analysis was undertaken to assess prognostic‐associated pyroptosis‐related lncRNA (prlncRNA) with statistically significant associations having *p*‐values <0.05. All the samples were randomly divided into training (*n* = 351) and validating sets (*n* = 148) randomly at a 7:3 ratio for further analysis. We performed LASSO regression to narrow down the prlncRNAs to construct the pyroptosis‐related lncRNAs prognostic signature in the training set. The coefficient of prlncRNA was determined by Cox proportional hazard regression analysis to compute the risk score for all patients using the algorithm:

$$\text{Risk score}=\sum_{i=0}^{n}\text{coefi}\times \text{prlncRNA expression}.$$

In the training set, we utilized the medium risk score as the threshold value for categorizing subjects of the training and validation sets into high‐ and low‐risk cohorts. The R packages utilized in these steps were “limma”, “pheatmap”, “survival”, “caret”, “glmnet”, “survminer”, and “timeROC”.

### 2.4 Validation of the constructed risk model

The risk curve and scatter plots were visualized using R software to show the survival status associated with the risk scores for individuals in both the training and validation set. We performed the KM survival curve of low‐ and high‐risk cohorts in the training set and validation set and compared the survival differences utilizing the Log‐rank test. After that, the independent prognostic significance of the risk model was assessed through univariate and multivariate Cox regression analysis. The R packages utilized in these steps included “survival”, “timeROC”, “survminer”, and “pHeatmap”. Principal component analysis (PCA) premised on the prlncRNAs signature expression was carried out to distinguish the low‐ and high‐risk cohort in all HNSCC patients using the “Rtsne” and “ggplot2” R packages. A multivariate operating characteristic curve (ROC) relative to the 1‐,2‐, and 3‐year survival for risk score and clinicopathological characteristics (including stage, age, grade, and sex) to predict the prognosis were generated using the “survival”, “survminer” “survivalROC”, and “timeROC” R packages. The area under the curve (AUC) was computed and contrasted to evaluate the efficacy of the prlncRNAs signature for predicting prognosis. To increase inspection efficiency, all HNSCC patients were used for subsequent analysis. Subgroup survival analysis was utilized to examine the usefulness of the risk model in each subgroup based on clinical stage, grade, age, N stage, sex, and T‐stage. To verify the clinical application value of the constructed model, the Pearson's chi‐squared test was utilized for contrasting the associations between the clinicopathological factors and risk model, and the results were illustrated in a heatmap. The Wilcoxon signed‐rank test was employed for comparison of the differences in risk score among various cohorts adjusted for these clinicopathological features.

### 2.5 Functional and pathway enrichment analysis

We employed the “DESeq2” package to identify differentially expressed genes (DEGs) between the low‐ and high‐risk cohorts according to specific criteria (|log2FC| ≥ 1 and FDR *q* < 0.05). After an initial screening, Gene Ontology (GO) and Kyoto Encyclopedia of Genes and Genomes (KEGG) enrichment analysis were executed on the identified DEGs utilizing the “clusterProfiler” and “ggplot2” packages. Moreover, Gene Set Enrichment Analysis (GSEA) was also employed to reveal the different signaling pathways activated in the low‐ and high‐risk cohorts having an FDR *q*‐values <0.05.

### 2.6 Tumor microenvironment and immune cell infiltration

Immune‐, stromal‐, and ESTIMATE scores in the tumor microenvironment (TME) for each HNSCC specimen were computed by applying the ESTIMATE algorithm utilizing the “estimate” package.^37^ The “CIBERSORT” package was implemented to infer the relative proportions of the 22 types of immune cells for each specimen premised on the CIBERSORT algorithm.^38^, ^39^ KM plotters were used to assess the potential prognostic effect of immune cells on HNSCC. Additionally, a single sample gene‐set enrichment analysis (ssGSEA) score was calculated to facilitate the quantification of tumor‐infiltrating immune cells (TIICs) and immune pathways for each sample, which was then compared to stratifying patients according to the low‐ and high‐risk cohorts premised on the 29 immune‐associated gene sets using “GSVA” package.^40^, ^41^

### 2.7 Immunotherapy and chemotherapy

The differential expression of an immune checkpoint inhibitor (ICI)‐related genes (*PD1*, *PD‐L1*, and *CTLA4*) between the low‐ and high‐risk cohorts were analyzed. The immunophenoscore (IPS) score of the TCGA‐HNSC project was obtained from The Cancer Immunome Atlas (TCIA) database (https://tcia.at/home) to assess the potential response to immunotherapy, which was computed premised on the gene expression values of immune‐related genes.^42^ The half‐maximum inhibitory concentration (IC50) of four commonly used chemotherapy drugs (paclitaxel, gemcitabine, docetaxel, and cisplatin) for HNSCC patients was estimated using the “pRRophetic” package.^43^

### 2.8 Statistical analysis

All statistical analyses and graphs were obtained by the R software (version 4.0.3). The chi‐squared test was utilized to examine the categorical variables whereas the Wilcoxon signed‐rank test was utilized in examining the continuous variables. For comparisons, Pearson's correlation analysis was utilized. A *p*‐value <0.05 denotes the statistical significance.

## 3 RESULTS

### 3.1 Expression and interaction of pyroptosis‐related genes in HNSCC patients

**FIGURE 1:**
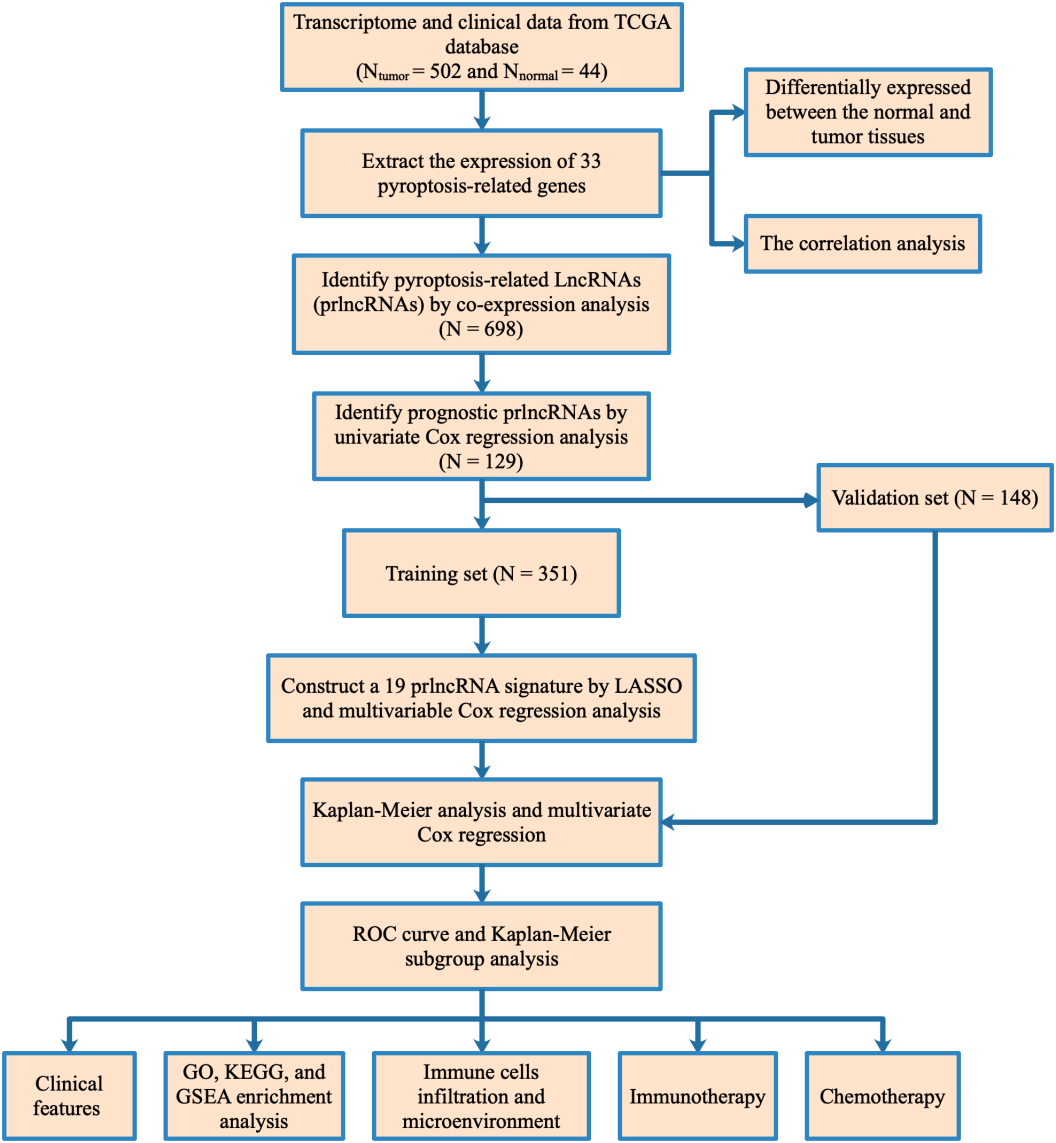
Flow diagram of the study. Abbreviations: GSEA, Gene set enrichment analysis; GO, Gene Ontology; KEGG, Kyoto Encyclopedia of Genes and Genomes; ROC, receiver operating characteristic; LASSO, Least Absolute Shrinkage and Selection Operator; TCGA, the cancer genome atlas; lncRNA, long noncoding RNA.

**FIGURE 2:**
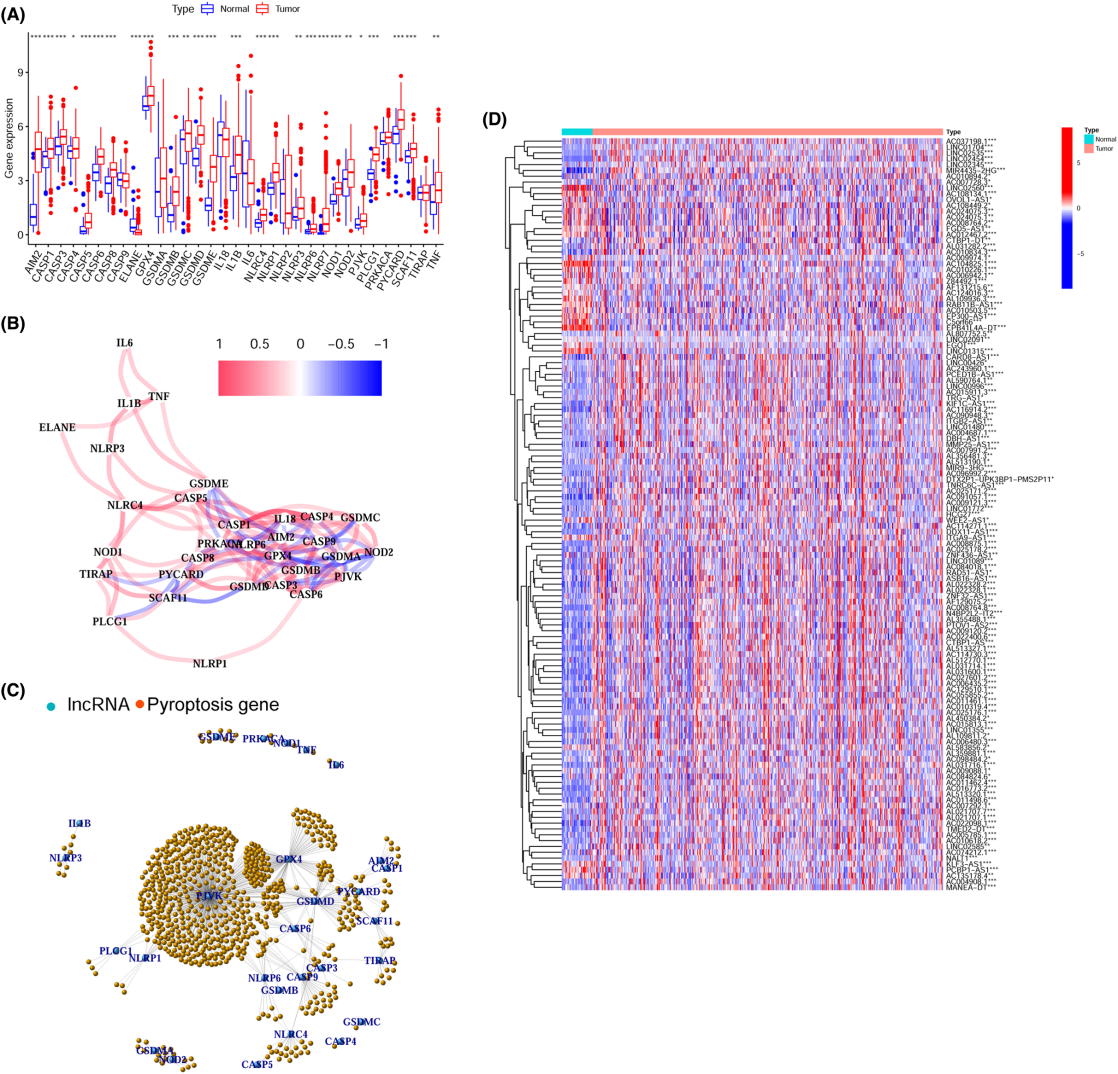
Identification of the pyroptosis‐related lncRNAs. (A) Comparison of expression of 33 pyroptosis‐related genes between the tumor and non‐tumor normal tissues. (B) The correlation network of the pyroptosis‐related genes (blue line: negative correlation, red line: positive correlation; the darker color reflects the strength of the relevance). (C) The correlation network of the pyroptosis‐related genes and pyroptosis‐related LncRNAs (orange dot: pyroptosis‐related gene, blue dot: pyroptosis‐related LncRNA). (D) Heatmap of the pyroptosis‐related LncRNAs between the normal and the tumor tissues. Blue signifies decreased expression while red signifies elevated expression. *p < 0.05, **p < 0.01, and ***p < 0.001.

Figure 1 describes the study flow. The expression of 33 pyroptosis‐related genes was compared in 502 HNSCC and 44 normal tissues from the TCGA database, and 26 DEGs were identified (for all *p* < 0.05). Among these, 25 genes (*AIM2*, *CASP1*, *CASP3*, *CASP4*, *CASP5*, *CASP6*, *CASP8*, *GPX4*, *GSDMB*, *GSDMC*, *GSDMD*, *GSDME*, *IL1B*, *NLRC4*, *NLRP2*, *NLRP3*, *NLRP6*, *NLRP7*, *NOD1*, *NOD2*, *PJVK*, *PLCG1*, *PYCARD*, *SCAF11*, and *TNF*) were highly expressed in HNSCC tissue and only *ELANE* showed higher expression in normal tissues (Figure 2A). Cross‐correlations between pyroptosis‐related genes according to Pearson's correlation analysis are shown in Figure 2B (the red line represents positive correlation whereas the blue line represents negative correlation; |Pearson *R*| > 0.2; *p* < 0.05).

### 3.2 Construction of the prognostic signature based on pyroptosis‐related lncRNAs

A total of 698 prlncRNAs were screened using Pearson's correlation analysis. The interaction network between pyroptosis‐related genes and prlncRNAs is shown in Figure 2C and Table S2. Of these, PJVK had the greatest number of related lncRNAs. Next, univariate Cox regression analysis was executed to identify 129 prognostic prlncRNAs among 499 HNSCC patients with the corresponding survival information, of which the comparison of gene expression in tumor and non‐tumor normal tissues is illustrated in Figure 2D. Premised on the ratio of 7:3, a randomization approach was used to allot an aggregate of 351 HNSCC patients to the training set and 148 patients to the validation set. Furthermore, 19 prlncRNAs were selected to establish the prognostic prlncRNA signature by LASSO analysis in the validation set. Finally, we performed multivariate Cox regression to calculate coefficients to develop the risk model (Table S3). Subsequently, the risk score for all samples was computed utilizing the aforementioned algorithm. Patients were stratified into two cohorts, namely low risk and high risk, premised on the training set's median risk score.

### 3.3 Evaluation of the prognostic value of the constructed prlncRNA signature

**FIGURE 3:**
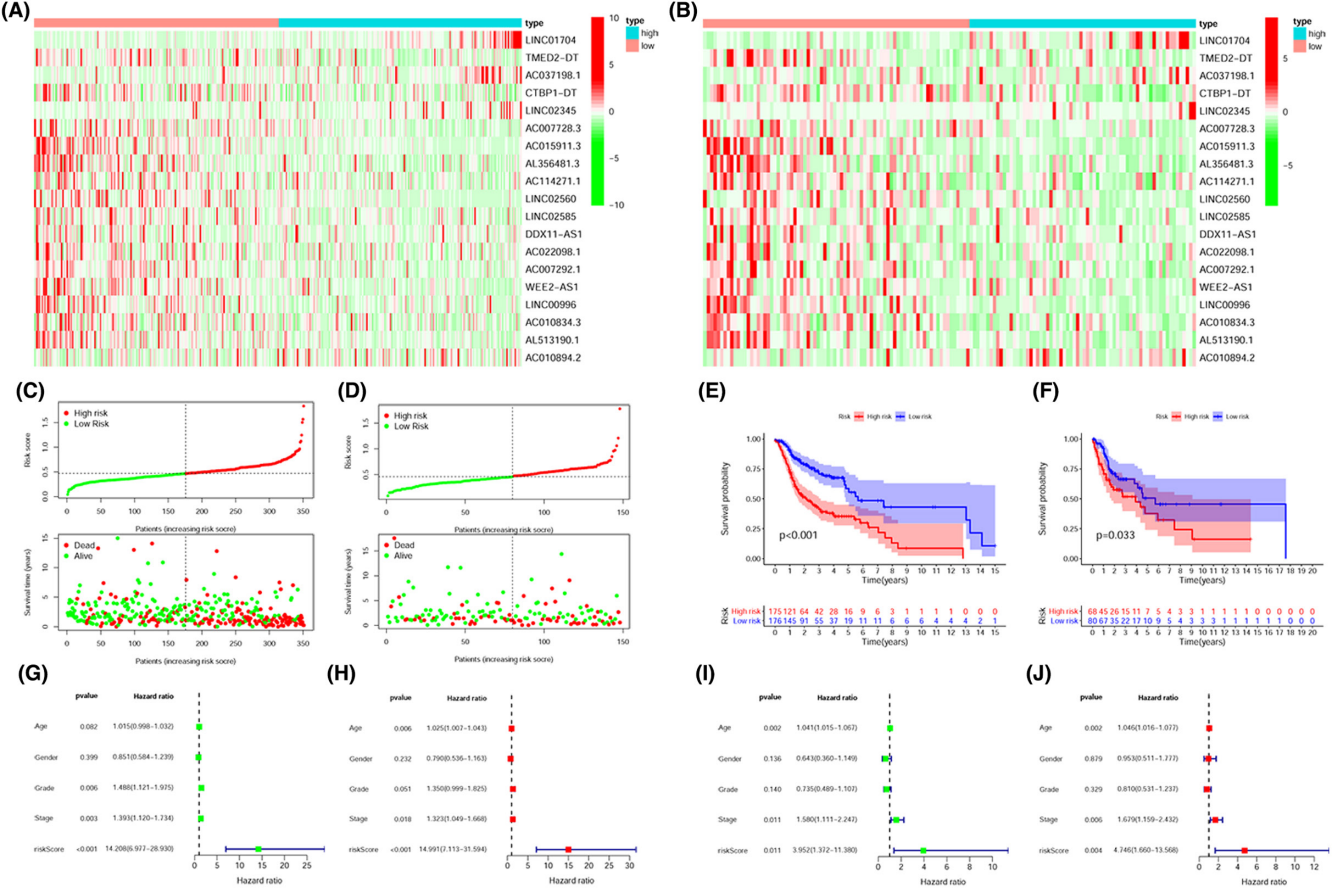
Construction of the pyroptosis‐related LncRNA prognostic signature. The heatmap showed the expression level of 19 pyroptosis‐related LncRNAs in the low‐ and high‐risk cohort in both the training (A) as well as the validation sets (B). The distribution and survival status of each patient premised on the median risk score (the left of the dotted line signifies the low‐risk cohort while the right side signifies the high‐risk cohort) in both the training (C) and validation set (D). KM survival analysis illustrated that the OS of the high‐risk cohort was substantially poor as opposed to the low‐risk cohort in both training (E, Log‐rank *p* < 0.001) and validation sets (F, Log‐rank *p* = 0.033). Univariate (G) and multivariate (H) Cox regression analysis of risk score in the training cohort. Univariate (I) and multivariate (J) Cox regression analysis of risk score in the validation cohort.

**FIGURE 4:**
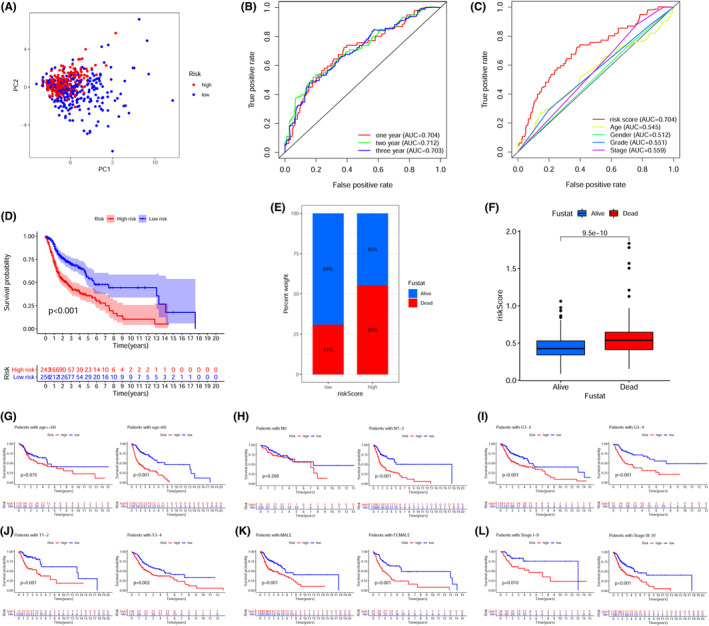
The prognostic significance of the pyroptosis‐related LncRNA signature risk score model was verified utilizing all TCGA‐HNSC patients. (A) PCA scatter plot for the expression of 19 pyroptosis‐related lncRNAs distinguishing the low‐ and high‐risk cohorts. (B) The ROC curve of the risk score model for 1‐, 2‐, and 3‐years overall survival. (C) ROC curve of the risk score and other clinicopathological parameters (age, sex, grade, and stage). (D) KM survival curves illustrated that the OS of the high‐risk cohort was worse as opposed to the low‐risk cohort (Log‐rank *p* < 0.001). (E) The survival proportions of patients in the high‐ and low‐risk cohort. (F) Relationship between the risk score and survival status. The subgroup analysis of Kaplan–Meier survival curve based on (G) age, (H) N‐stage, (I) histopathological grade, (J) T‐stage, (K) sex, and (L) clinical stage.

**FIGURE 5:**
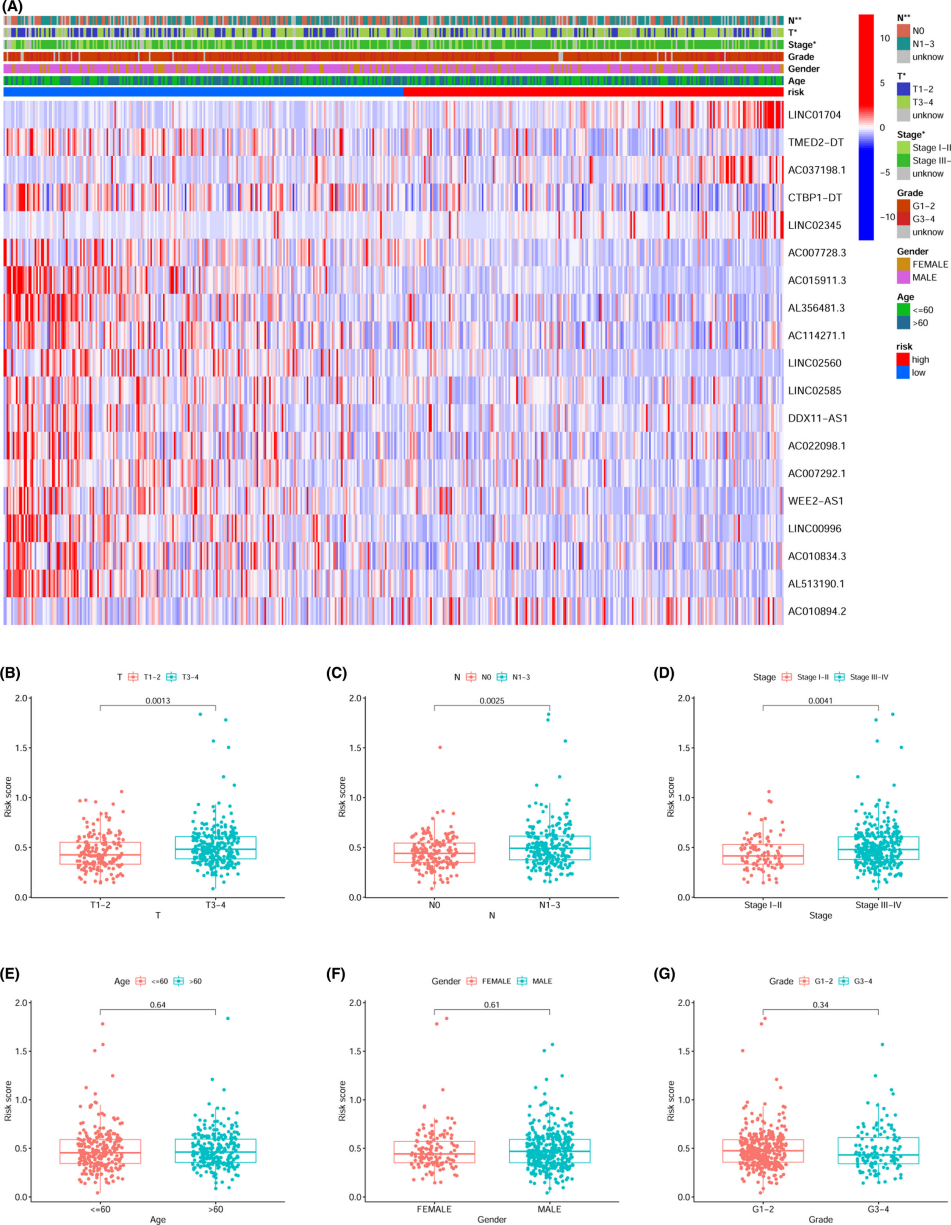
Association between the clinicopathological data and risk score of patients with HNSCC. (A) Heatmap showed clinicopathologic features of the high and low‐risk cohorts. The boxplot showed the association between the risk score of the (B) T‐ stage, (C) N‐stage, (D) clinical stage, (E) age, (F) sex, and (G) histopathological grade.

The expression of 19 prlncRNAs between the low‐ and high‐risk cohorts was shown in both the training (Figure 3A) as well as the validation sets (Figure 3B). We observed that the majority of prlncRNAs had higher expression in the low‐risk cohort. The scatter plots demonstrated that patients in the low‐risk cohort exhibited extended periods of survival and lower mortality rates as opposed to those in the high‐risk cohort in both the training (Figure 3C) as well as the validation sets (Figure 3D). Furthermore, in the training set, KM analysis confirmed that the patients with high‐risk scores exhibited a considerably worse prognosis as opposed to those having low‐risk scores. (Figure 3E, *p* < 0.001). Consistent with the results in the training set, the survival curves illustrated that the high‐risk cohort had a poorer prognosis as opposed to the low‐risk cohort in the validation set (Figure 3F, *p* = 0.033). Multivariate and univariate Cox regression analysis showed the risk score was an independent prognostic indicator in the training set (Figure 3G: univariate HR = 14.208; Figure 3H: multivariate HR = 14.991, *p* < 0.05), which was also verified in validation set (Figure 3I: univariate HR = 3.952; Figure 3J: multivariate HR = 4.746, *p* < 0.05). To improve the reliability of the score, all samples were included in the subsequent analyses. PCA plots showed the satisfactory separation between low‐ and high‐risk cohorts (Figure 4A). The AUC of individual ROC curves of risk scores (Figure 4B) associated with 1‐, 2‐, and 3‐years survival were 0.704, 0.712, and 0.703, in that order. Figure 4C indicated a more reliable predictive ability of OS of risk score when contrasted with other clinical features including sex, age, tumor grade, and tumor stage). The KM survival plot and bar plots indicated that the low‐risk patients achieved longer survival times (Figure 4D, Log‐rank *p* < 0.001) and lower death rates compared to the high‐risk cohort (Figure 4E, 31% _VS._ 55%). It is logical that the risk score was considerably elevated in patients who had died compared to surviving patients (Figure 4F, *p* < 0.001). With regards to the subgroup analysis, the prlncRNA prognostic signature adjusted for age (Figure 4G) and N‐stage (Figure 4H), showed no survival differences for age ≤60 and the N0 subgroup. However, the signature was effective for stratifying other subgroups based on the tumor grade (Figure 3I), T‐stage (Figure 4J), sex (Figure 4K), and clinical stage (Figure 4L). To explore the clinical application value of the prlncRNA signature, a heatmap (Figure 5A) illustrating the association between the risk model and clinicopathological characteristics was constructed. The chi‐squared test showed the N‐stage, T‐stage, and clinical stages were considerably correlated with the risk model (all *p* < 0.05). Further analysis revealed that the risk score was considerably elevated in the T3‐4 (Figure 5B, *p* = 0.001), N1‐3 (Figure 5C, *p* = 0.003), and stages III‐IV (Figure 5D, *p* = 0.004) subgroups. However, there were no differences between the risk score and adjustments for age (Figure 5E), sex (Figure 5F), or tumor grade (Figure 5G).

### 3.4 Functional enrichment analysis

**FIGURE 6:**
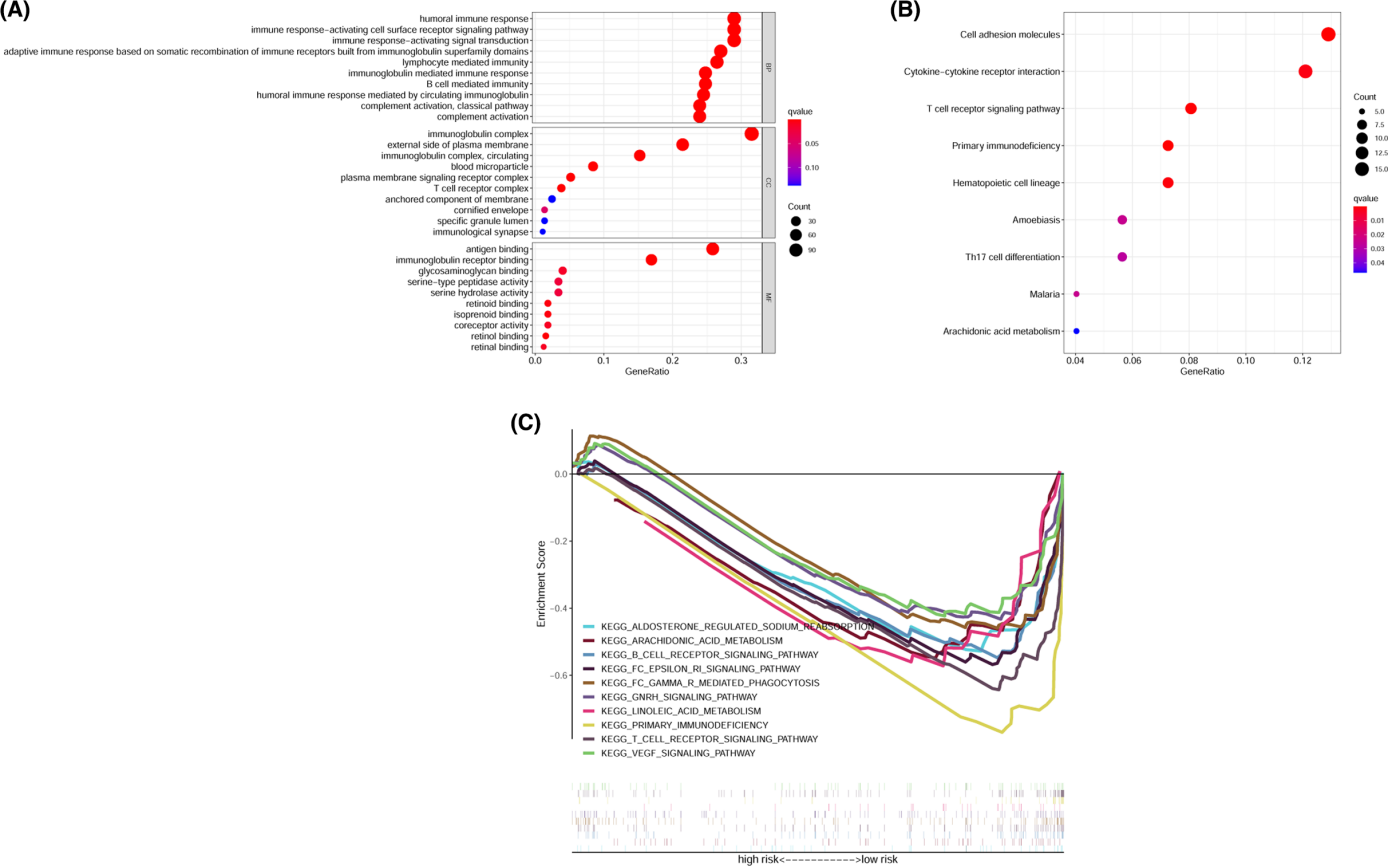
Functional enrichment analysis between low‐ and high‐risk cohorts. (A) GO analysis (B) KEGG analysis, and (C) Gene Set Enrichment Analysis (GSEA) analysis between the low‐ and high‐risk cohorts.

A total of 499 DEGs were identified between the low‐ and high‐risk cohorts (including 24 upregulated DEGs and downregulated 475 DEGs) (Table S4). GO enrichment analysis (Figure 6A) of DEGs showed the involvement of several immune‐related biological processes including humoral immune response, B cell‐mediated immunity, humoral immune response mediated by circulating immunoglobulin, lymphocyte‐mediated immunity, adaptive immune response premised on somatic recombination of immune receptors constructed from immunoglobulin superfamily domains, immune response activating signal transduction, immune response activating cell surface receptor signaling pathways, and the complement activation classical pathway. KEGG pathway analysis (Figure 6B) of the DEGs also revealed several immune‐related pathways including the T‐cell receptor signaling pathway, cytokine–cytokine receptor interaction, and the primary immunodeficiency pathway. GSEA enrichment analysis (Figure 6C) revealed there was no considerable enrichment of pathways in the high‐risk cohort, but demonstrated a considerable enrichment of immune‐associated and cancer‐associated pathways in the low‐risk cohort including the Fc gamma R‐mediated phagocytosis, Fc epsilon RI signaling pathway, B cell receptor signaling pathway, primary immunodeficiency, T‐ cell receptor signaling pathway, and VEGF signaling pathway.

### 3.5 Association between the risk score and the TME, as well as immune cells infiltration

**FIGURE 7:**
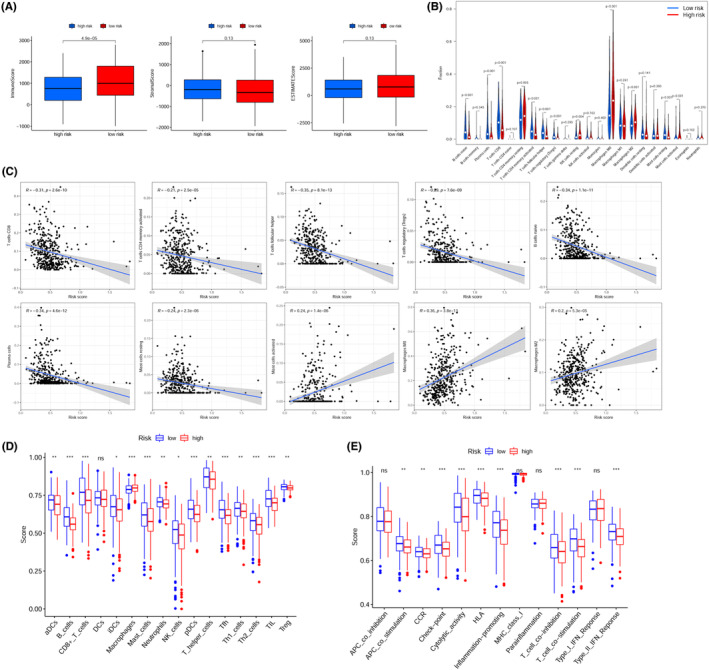
Relationship between the risk score and components of the TME, and TIICs. (A) Distribution of risk scores stratified by immune, stromal, and ESTIMATE scores calculated using the ESTIMATE algorithm. (B) The comparison of the fractions of immune cells between low‐ and high‐risk cohorts was calculated using the CIBERSORT algorithm. (C) Pearson's correlation analysis of the risk score and infiltrating immune cells (*R* > 0.2, *p* < 0.05). (D) The comparison of the ssGSEA enrichment scores of 16 types of immune cells between the low‐ and high‐risk cohorts. (E) Comparison of the ssGSEA enrichment scores of 13 immune‐related pathways between low‐ and high‐risk cohorts.

The TME consisted of immune cells, stromal cells, and soluble factors, as well as cancer cells. The immune scores within the low‐risk cohorts were considerably elevated as opposed to those in the high‐risk cohort (Figure 7A, *p* < 0.001), implying that the low‐risk cohort exhibited a greater level of immune cell infiltration as opposed to the high‐risk cohort. The association of the risk model and immune cells infiltration was also analyzed in HNSCC patients. Figure 7B,C showed that the proportion of regulatory T cells (Treg), CD8+ T cells, activated CD4+ T cells, follicular helper T cells (Tfh), plasma cells, resting mast cells, and B cells increased in the low‐risk cohort but showed strong negative correlation with the risk score. However, activated mast cells, M0 macrophages, and M2 macrophages were activated in the high‐risk cohort and were positively linked to the risk score. Furthermore, KM survival analysis (Figure S1) showed that activated mast cells (*p* < 0.001), M0 macrophages (*p* = 0.041), and M2 macrophages (*p* = 0.023) were associated with poor prognosis of HNSCC based on OS, conversely, regulatory T cells (*p* = 0.018), follicular helper T cells (*p* = 0.038), plasma cells (*p* = 0.002), resting mast cells (*p* < 0.001), and B cells (*p* < 0.001) were concerned with favorable prognosis, which might partially explain the worse prognosis of high‐risk cohort. Except for macrophages, the low‐risk cohort exhibited an elevated ssGSEA score of dendritic cells (total DCs, activated DCs, immature DCs, and plasmacytoid DCs), mast cells, neutrophils, natural killer (NK) cells, CD8+ T cells, T helper (Th) cells (Tfh, Th1, and Th2 cells), B cells, tumor‐infiltrating lymphocytes (TILs), and Tregs as opposed to the high‐risk cohort (Figure 7D). Additionally, except for the type II IFN response pathway which was found to be enriched in the high‐risk cohort, ssGSEA analysis illustrated that eight immune functions were increasingly activated in the low‐risk cohort (Figure 7E).

### 3.6 Correlation between risk scores and immunotherapy, and potential efficacy of chemotherapeutics for HNSCC patients

**FIGURE 8:**
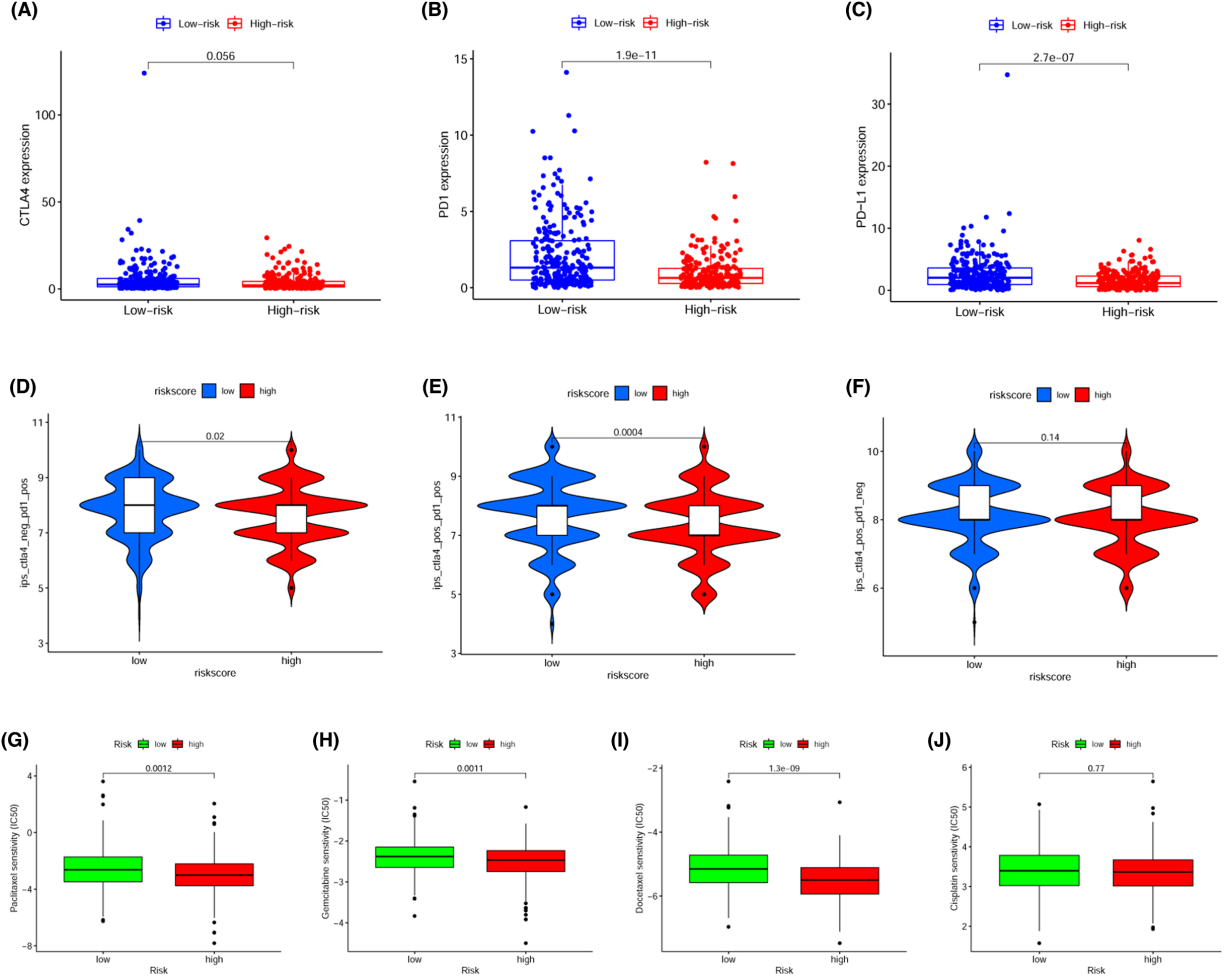
Evaluation of the correlation between risk scores and potential response to immunotherapy and chemotherapeutics for HNSCC patients. (A) The link between the risk score and expression of (A) CTLA4, (B) PD1, and (C) PD‐L1. The association between the risk score and the Immunophenoscore (IPS) of anti‐PD1 monotherapy (D), the combination of anti‐PD1 and anti‐CTLA4 immunotherapy (E), and anti‐CTLA4 monotherapy (F). Drug sensitivity analysis of (G) paclitaxel, (H) gemcitabine, (I) docetaxel, and (J) cisplatin for HNSCC patients at the high‐ and low‐risk cohorts.

Except for *CTLA4* (Figure 8A, *p* = 0.056), the expression of *PD1* (Figure 8B, *p* < 0.001), and *PD‐L1* (Figure 8C, *p* < 0.001) were remarkably elevated in the low‐risk cohort as opposed to those in the high‐risk cohort, implying that the low‐risk patients might achieve a better response to ICIs. The violin plots based on the IPS validated the above results, suggesting that low‐risk patients would achieve a better response to either PD‐1 inhibitor treatment alone (Figure 8D, *p* = 0.02) in combination with a CTLA4 inhibitor (Figure 8E, *p* < 0.001), while for these patients' treatment with CTLA4 inhibitor alone was not suitable (Figure 7F, *p* = 0.14). The IC50 of each HNSCC patient was estimated based on the pRRophetic algorithm. A significantly lower IC50 in the high‐risk cohort was observed for paclitaxel (Figure 8G, *p* = 0.001), gemcitabine (Figure 8H, *p* = 0.001), and docetaxel (Figure 8I, *p* < 0.001), whereas for cisplatin (Figure 8J, *p* = 0.77), no difference was identified between the low‐ and high‐risk cohorts, indicating that the high‐risk cohort had a higher sensitivity to paclitaxel, gemcitabine, and docetaxel treatment.

## 4 DISCUSSION

HNSCC patients exhibit considerable heterogeneity in biological behavior, therapeutic response, and prognosis; thus, commonly used prognostic factors, such as age, grade, and TNM classification, are not suitable for accurate prognostic prediction.^44^ Increasing evidence has shown that pyroptosis is a key mechanism in cancer progression and progressive chemosensitization.^45^ Moreover, previous studies have provided growing evidence that lncRNAs are stable and tissue specific, making them auspicious biomarkers for early diagnosis as well as improved prognosis of illnesses.^46^ Therefore, it would be useful to establish a pyroptosis‐related lncRNA signature for prognostic prediction for HNSCC patients.

Premised on the TCGA dataset, we identified 19 prognostic‐associated prlncRNAs to establish a novel pyroptosis‐related prognostic lncRNAs signature utilizing the LASSO analysis and COX regression analysis in the training set. KM survival analysis and multivariate Cox regression were employed to verify that the risk model illustrated that prlncRNA signature might function as an independent prognostic indicator for HNSCC patients in both the training as well as the validation sets. Additionally, multivariate ROC curves and Kaplan–Meier survival analysis using the total sample population confirmed the risk model could achieve a more efficient predictive performance compared with other conventional prognostic factors (age, sex, grade, and stage). Further subgroup analysis premised on clinical stage, age, N stage, sex, grade, and T‐stage showed that the prognostic signature was effective in all subgroups except for age ≤60 and the N0 subgroup and indicated the universality of this prognostic signature. Meanwhile, the chi‐squared test and the Wilcoxon signed‐rank test confirmed that the risk model was remarkably correlated with T‐stage, N‐stage, and the clinical stage of HNSCC patients. Altogether the above analyses confirmed that this prlncRNA signature was highly reliable and effective for prognosis prediction of HNSCC patients.

The GO and KEGG analysis illustrated that the DEGs between the low‐risk and high‐risk cohorts were related to immune‐associated biological pathways and processes. Consistent with these findings, the GSEA analysis also illustrated the enrichment of immune‐associated pathways in the low‐risk cohort. This is a logical result as pyroptosis is closely associated with immune activation and inflammation.^47^, ^48^ The TME comprises non‐cancer cells surrounding tumors and the infiltration of the immune cells in the TME performs a crucial function in the occurrence, development, as well as metastasis of tumors via paracrine signaling or physical factors.^49^, ^50^, ^51^ We determined that immune scores were considerably elevated in the low‐risk cohort as opposed to the high‐risk cohort by the ESTIMATE algorithm, which indicated that greater immune cell infiltration within the low‐risk cohort. Further, the analysis of immune cells infiltration utilizing CIBERSORT and the ssGSEA algorithm showed that abundant antitumor infiltrating immune cells (NK cells, B cells, T‐helper cells, CD4+ T cells, and CD8+ T cells) and eight immune pathways were enriched in the low‐risk cohort. Both CD4+ T cells, as well as the CD8+ T cells, are the main cells that participants in the immune response and are active in the antitumor response,^52^, ^53^ which could explain the improved prognosis for in cancer patients.^54^, ^55^ Interestingly, consistent with the pyroptosis‐related gene signature in ovarian cancer constructed by Ye et al.^56^ the low‐risk cohort had significantly higher proportions of Treg cells than the high‐risk cohort. It is generally thought that Treg cells suppress antitumor immune responses by impairing cell‐mediated immune responses to tumors and thus, correlate with poorer clinical outcomes.^57^ However, several studies found that Treg cells display functional instability and heterogeneity in different cancer types.^58^, ^59^ Meanwhile, functionally distinct subtypes of Treg cells have opposite roles in determining cancer prognosis.^60^ Thus, we suspected that abundant non‐suppressive Treg cells might be recruited by the inflammatory reactions of pyroptosis in the low‐risk cohort. Moreover, M2 macrophages demonstrated higher infiltration in the high‐risk cohort as opposed to the low‐risk cohort, and are well known as potent immunosuppressive cells associated with relapse, distant metastasis, and poor outcome in several cancers.^61^, ^62^ According to the above findings, we have reason to believe that immune evasion and dysfunction may result in poorer outcomes in the high‐risk cohort.

Many novel immunotherapeutic strategies, including the approved ICIs, chimeric antigen receptor T‐cell therapy, bispecific antibodies, and dendritic cell vaccination, have greatly improved the clinical outcome of cancer patients.^63^, ^64^, ^65^ Considering the recent approval of anti‐PD‐1/PD‐L1 antibodies (pembrolizumab and nivolumab) for HNSCC as a first‐line treatment in recurrent and metastatic disease by the United States of America Food and Drug Administration, new treatment options for HNSCC are available and have shown promising results.^66^, ^67^ However, only a minority of patients is responsive to ICIs and achieve durable benefits, the identification of predictive markers and the mechanisms involved in resistance to immunotherapy are the key to widespread clinical application. Accumulating evidence has shown that pyroptosis evoked by antitumor immunity and precise modulation of pyroptosis might provide an important opportunity to improve the efficacy of immunotherapy.^68^ In our study, due to the strong association between the prlncRNA signature and immune cell infiltration, we investigated the association between the prlncRNA signature and immunotherapy in HNSCC patients. We discovered that the *PD‐L1* and *PD‐1* expression were substantially elevated in the low‐risk cohort as opposed to the high‐risk cohort, which meant that low‐risk patients might achieve improved responses to treatment with anti‐ PD‐L1/PD‐1 therapy.^69^ Further analysis based on the TCIA database confirmed that low‐risk patients would likely show a better response to PD‐1 inhibitor treatment alone or combined with the CTLA4 inhibitor than high‐risk patients, which is helpful to develop individualized and precise immunotherapy programs.

It has been accepted that apoptosis is the main form of cancer cell programmed cell death induced by chemotherapy.^70^, ^71^ However, pyroptotic cell death has recently been proposed as a new molecular mechanism responsible for the efficacy of various antitumor agents, including chemotherapeutic agents.^16^ Moreover, Zhang et al. reported that both paclitaxel, as well as cisplatin, could trigger the caspase‐3/GSDME signaling pathway and convert the cell death pathway from apoptosis to pyroptosis, in order to suppress the proliferation of lung cancer cells.^72^ In gastric cancer cells, 5‐fluorouracil‐induced caspase‐3 activation as well as the cleavage of GSDME; thus, switching chemotherapy drug‐induced caspase‐3‐dependent apoptosis into pyroptosis.^73^ In addition, Wu et al. found that cisplatin could promote the function of caspase‐3, contribute to pyroptosis of esophageal cancer cells, and in parallel enhance DNA damage.^74^ These findings provide new insight supporting the induction of caspase‐3‐dependent pyroptosis of cancer cells with chemotherapeutic drugs that will achieve improvements in cancer treatment. In the current study, we identified an association between the prlncRNA signature and chemosensitivity in HNSCC patients. In contrast to immunotherapy, our findings indicated that the high‐risk cohort would show greater sensitivity to gemcitabine and docetaxel treatment as opposed to the low‐risk cohort, indicating pyroptosis induced by chemotherapeutic drugs was more frequently observed in high‐risk patients and the prlncRNA signature might serve as a potential prognosticator for chemosensitivity of HNSCC.

The present study has some limitations that should be considered. Above all, the training set and validation sets were both obtained from TCGA, and the results would be more reliable if there were external validation cohorts (GEO datasets) for analysis available. Moreover, we did not explore the potential involvement of pyroptosis‐related lncRNA mechanisms on immunotherapy and chemotherapy for HNSCC and this question deserves further in‐depth studies. Additionally, more evidence is needed to support the role of 19 prlncRNAs in HNSCC. Furthermore, the significance of pyroptosis‐related genes in the HNSCC were still unexplored. Therefore, large‐scale, multicenter, and prospective studies are necessary to confirm our results in the future.

## 5 CONCLUSION

Overall, we successfully established and validated a pyroptosis‐related lncRNA signature that could independently predict the OS of HNSCC patients. In addition, we provided evidence that pyroptosis‐related lncRNA signature was linked to the TME and immune cell infiltration in HNSCC. Furthermore, this signature might predict the immunotherapeutic response and chemosensitivity of HNSCC patients, which contribute to individualized, accurate, and precise treatment decisions.

## AUTHOR CONTRIBUTIONS

The research was designed by CZ and HD. The data analysis from the public database was made by CZ, YJ, MT, ZS, and DY. The data were analyzed by CZ, ZS, and YS. HD supervised the writing of the draft. The manuscript was reviewed by all the authors.

## CONFLICT OF INTEREST

The authors state that the study was carried out without any commercial or financial relationships that might be seen as a possible conflict of interest.

## ETHICS STATEMENT

All data of this study were public and required no ethical approval.

## Supporting information


Table S1

Table S2

Table S3

Table S4

## Data Availability

The data used for our analysis in this study are openly available from The Cancer Genome Atlas at https://portal.gdc.cancer.gov/.
